# Clinical Interpretation of Genomic Variations

**DOI:** 10.4274/tjh.2016.0149

**Published:** 2016-08-19

**Authors:** Müge Sayitoğlu

**Affiliations:** 1 İstanbul University Faculty of Medicine, Institute of Experimental Medicine, Department of Genetics, İstanbul, Turkey

**Keywords:** Genetic variation, Sequencing, Genomic data, Clinical interpretation

## Abstract

Novel high-throughput sequencing technologies generate large-scale genomic data and are used extensively for disease mapping of monogenic and/or complex disorders, personalized treatment, and pharmacogenomics. Next-generation sequencing is rapidly becoming routine tool for diagnosis and molecular monitoring of patients to evaluate therapeutic efficiency. The next-generation sequencing platforms generate huge amounts of genetic variation data and it remains a challenge to interpret the variations that are identified. Such data interpretation needs close collaboration among bioinformaticians, clinicians, and geneticists. There are several problems that must be addressed, such as the generation of new algorithms for mapping and annotation, harmonization of the terminology, correct use of nomenclature, reference genomes for different populations, rare disease variant databases, and clinical reports.

## Abstract (tr)

Yeni dizileme teknolojileri, tek genli ve/veya kompleks kalıtılan hastalıklarla ilgili genlerinin haritalanması, kişiye özel tedavi ve farmakogenomik alanları için yüksek verimde ve büyük ölçekte genomik data üreten teknolojilerdir. Yeni nesil dizileme, hastaların tanı ve tedavi yanıtlarını değerlendirecek moleküler izlem süreçlerinde kullanılmak üzere, hızlı bir şekilde rutin uygulamada yerini bulmaktadır. Yeni nesil dizileme platformları çok büyük ölçekte genetik varyasyon datası üretmektedir ve bu varyasyonların klinik olarak anlamlandırılması çok zordur. Klinik yorumlamalar, hekimler ile biyoinformatik ve genetik uzmanlarının yakın işbirliğine ihtiyaç duymaktadır. Yeni haritalama ve hizalama araçlarına duyulan ihtiyaç, terminolojinin harmonizasyonu, genetik isimlendirmenin doğru kullanımı, farklı populasyonlar için referans genom datasının bulunmaması, nadir hastalıklar için genomik veri bankalarının eksikliği ve klinik raporlama, halen aşılması gereken sorunlar arasında bulunmaktadır.

## INTRODUCTION

Next-generation sequencing (NGS) methods provide cheap and solid genomic data and are used extensively for de novo sequencing, disease mapping, quantifying of expression levels, and population genetic studies [1,2,3,4,5]. They can also be applied to complex disorders [6], personalized treatment, and pharmacogenomics [7,8,9]. The medical genetics field translates high-throughput genetic data to clinical settings in order to improve diagnostic efficiency and treatment decision-making [10,11]. The interpretation of the clinical significance of genomic variants in a given patient or in patients’ family members is the main challenge of resequencing. In the last decade, several diseases and syndromes have been analyzed by NGS, hundreds of disease-associated genes have been found, and novel targeted therapies have been developed. The most powerful contribution of NGS [particularly whole-exome sequencing (WES) and whole-genome sequencing (WGS)] is the description of new candidate signaling pathways involved in the pathogenesis of a clinical condition that will help with prevention, diagnosis, and therapeutic opportunities.

High-throughput sequencing can be implemented within different applications including WGS, WES, ribonucleic acid sequencing (RNA-seq), or targeted sequencing [12]. Commercially available NGS platforms are generally employed with similar steps for all these approaches: generation of sequencing libraries, sequencing simultaneously in a massively parallel fashion, and data analysis [12].

Whole-exome sequencing studies have been commonly used to identify the responsible gene of a clinical phenotype. The WGS approach holds major advantages for the detection of variations not only in the exome but also in the noncoding regulatory regions for complex and/or multigenic diseases. The analysis of WGS data is complicated by the amount of information and challenges in elimination of common genetic variations. Whole-exome sequencing studies require rough bioinformatics analysis work and experts and national reference sequences to evaluate the population-based genetic variants, along with large budgets.

Alternatively, WES is relatively cost-efficient and is able to discover disease-related rare variants in coding regions and splice sites. There are several variations that have been successfully identified by WES in monogenic diseases. On the other hand, the exome represents less than 1% of the genome and such analysis will be excluding noncoding genomic regions such as regulatory regions, repetitive sequences, or noncoding RNAs.

Using gene panels in NGS studies is an alternative option that restricts screening to selected genetic regions. Although it may simplify the scale of the analysis and interpretation, incidental findings still require attention. The most suitable NGS approach for routine clinical applications is amplicon-based/targeted sequencing. Most genetic disorders have allelic and locus heterogeneity, which means that one disease may arise from different genetic variations within the same gene or different genes. Due to the genetic heterogeneity, it takes longer to obtain the genetic test result, which leads to a delay in diagnosis. Amplicon-based NGS has provided a major advantage for the molecular analysis of heterogeneous genetic disorders, including hereditary cancers [13].

RNA-seq quantifies the amount of transcripts (all transcribed isoforms) and gives a chance to evaluate the whole RNA repertoire of a specific cell or tissue. The biggest limitation of RNA-seq is the “noncoding RNAs”; most of the genome is transcribed but the majority of these transcripts are not translated into proteins [14].

## ACCURATE USE OF THE TERMINOLOGY: IS IT A POLYMORPHISM OR A DISEASE-RELATED VARIATION?

In common use, a DNA polymorphism is a heritable variation that is present in >1% of the population and increasingly detected by next-generation resequencing. One of two or more alternate forms of a locus (alleles) may result from the changes in the nucleotide sequence [single nucleotide polymorphisms (SNPs)], deletions, insertions, or other structural rearrangements. According to the novel terminology the term ‘SNP’ is used as single nucleotide variation (SNV) [15]. A genome contains repetitive sequences differing in copy numbers (i.e. copy number variations) between individuals. Polymorphic variations may or may not have phenotypic effects and they are valuable tools for genetic mapping studies including linkage and association studies of diseases. The vast majority of these variations (more than 90%) have been found to be localized in the noncoding genomic regions and are possibly involved in regulation of gene expression. On the other hand, a mutation is defined as a DNA variant detectable in <1% of the population and generally having phenotypic consequences.

The alternative use of the terms of “polymorphism” and “mutation” for an event (a difference compared with a reference standard) commonly leads to misinterpretation. This problem also can affect the accuracy of clinical interpretation and the functional relationship between a phenotype/disease and a genomic sequence. It is critical to establish clear nomenclature and guidelines regarding the identified genomic variations and their reporting. The definition of a “genetic variant” is currently in use to describe differences in comparison to a reference standard. This term can include neutral, benign, functional, pathogenic, deleterious, damaging, disease-associated, or causal definitions. Uniform terminology is recommended to correct the interpretation of a variant and to share correct genomic information. The Human Genome Variation Society (HGVS; http://varnomen.hgvs.org/) established a standard gene variant nomenclature and it is recommended for use as the primary guideline for determining variants [16]. Each genome has nearly 4 million genetic variations and each exome covers nearly 13,000 SNVs. The challenge facing researchers and clinicians is to depict the biological and clinical significance of these variants and transfer this information to clinical practice.

**Figure 1:**
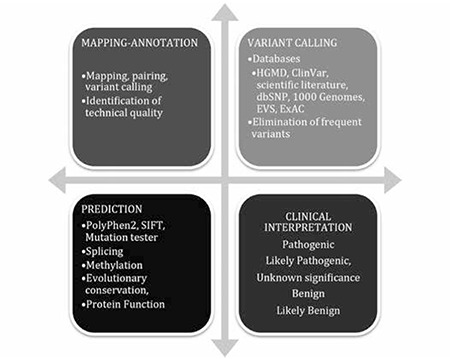
Main steps of re-sequencing data analysis.

Basic resequencing data analysis includes base calling, mapping, variant calling, and annotation steps (Figure 1). Every step in the variant interpretation process has limitations and difficulties including variation type, sample source and quality, and clinical heterogeneity, among other factors.

## 1) BASE CALLING (IMAGE PROCESSING)

Next-generation sequencing platforms are able to generate millions of reads to reduce the costs. However, despite the technological progress, NGS results are adversely affected by biochemical and signal acquisition mistakes. Next-generation sequencing platforms have different performance levels that rely on complex interactions of the chemistry, the hardware, and the optical sensors that they use. For example, in the Illumina system, the images that are acquired from the instruments are prepared and analyzed to determine the base incorporated in the complementary strand. In this process, the ordering of nucleotides in a template from the noisy signals is referred to as base calling [17]. In other words, base calling converts the fluorescence signals into actual sequence data with quality scores. Base calling accuracy is measured by a Q score (Phred quality score), which is a common metric to assess the accuracy of a sequencing run. The Q score is defined as the logarithmically related base calling error probability (Q=-10 log P/log 10) [18]. For example, if Q=40 for a sequencing run, this is equal to the probability of an incorrect base call of 1 in 10,000 times, or with 99.99% base calling accuracy or a lower Q score of 10 means, there is the probability of an incorrect call in 1 of 10 bases. Low Q scores lead to false positive variant calls and need resequencing.

Errors arising from NGS data are generally due to base calling and alignment applications. Moreover, low coverage sequencing (<5×) includes the high probability that only one of the two chromosomes of a diploid individual has been sampled at a specified site. Another option to improve base calling accuracy could be to increase the sequencing cycles (coverage=resequencing copies). Several base calling strategies haveü been developed to infer the correct base more reliably and to perform base calling faster [19].

## 2) MAPPING (ALIGNMENT TO A REFERENCE GENOME)

After successful base calling, the next step is the mapping or alignment of the sequenced genomic region. The main challenge is to accurately find the true location of each read from a large quantity of reference data and then to distinguish the technical sequencing errors and disease-related genetic variation within the sample. Next-generation sequencing platforms generally produce short reads (~200-300 bp) and we need to align or map these fragments to a reference sequence to find the corresponding part of the short reads. There are some limitations to mapping: 1) Reference sequences can be very long; for example, it is ~3 billion bases for humans and it is a crucial task to find the matching short region. 2) Since the sequences are short they can align to several places that have similar DNA sequences (such as repetitive sequences) in the genome. 3) It is not possible to get a perfect alignment because of in/del variations in the genome, so there will be some mismatches or gaps during the mapping.

Mappers perform global or local alignments with respect to the approach; for example, WGS and WES data need appropriate reference sequences to find all the genetic variations. RNA sequencing data can be mapped to the full reference sequence, or to a special transcriptome reference. Mapping necessitates computational time and critical computational requirements. There are several tools (e.g., BWA, SAM, GATK (http://www.broadinstitute. org/gatk/), Bowtie, or RMAP) that are available and designed for aligning DNA, RNA, or proteins [20,21,22,23,24].

## 3) VARIANT CALLING

Variant calling is an important procedure for resequencing deep sequencing analysis. Next-generation sequencing platforms generate huge amounts of genetic variation data; the main challenge is to discriminate a small subset of functionally important variants. When analyzing WES or WGS data, comparison with a correct reference plays a pivotal role. Determining the genetic variation of a reference genome from the target genome allows the identification of the disease-related genetic variations. Variant calling predicts the nucleotide differences versus a reference sequence (genome or transcriptome) at a given position, generally accompanied by an estimate of variant frequency and confidence intervals. Integrity of the alignment has a crucial role in variant detection; if the sequence is incorrectly aligned, it may lead to spontaneous errors in variant calling.

Genomic variants, such as SNVs, insertions, deletions, and in/dels (the occurrence of an insertion and deletion at the same time) can be identified by various analysis pipelines [25]. “SNV calling” identifies single nucleotide variable sites, whereas “genotype calling” determines the genotype for each individual at each site. To reduce the uncertainty associated with SNV calling is to increase the coverage (at >20× coverage). In association studies, sequencing many individuals at a low depth, rather than sequencing fewer individuals at a high depth, could maximize mapping power. Some of the genomic regions are difficult to interpret, such as homopolymer regions (a sequence of identical bases, like AAAA or TTTTTTTT), or simple repeats (minisatellite-variable number of tandem repeats and microsatellite-short tandem repeats). Bioinformaticians use VCF files, “Variant Call Format”, to store the gene sequence variations.

## 4) ANNOTATION AND PRIORITIZATION OF A VARIATION

Several challenges arise for NGS-based diagnostic and research efforts in the identification of all genetic variations. Because of the increased complexity of data analysis and clinical interpretation of the data, it is best to use some universally accepted recommendations like those of the American College of Medical Genetics and Genomics (ACMG), EuroGentest, and the European Society of Human Genetics [25,26,27].

**Figure 2:**
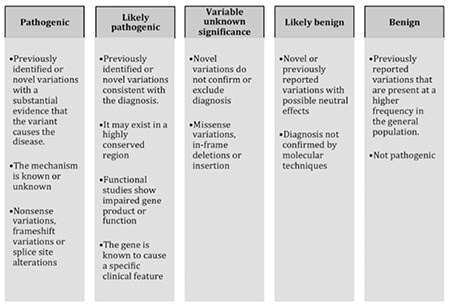
Recommended terms for interpretation of clinical variants.

The ACMG recommends that both “mutation” and “polymorphism” can be replaced by “variant” with the following modifiers: pathogenic (P), likely pathogenic (LP), variant of uncertain significance (VUS), likely benign (LB), and benign (B) [26] (Figure 2). The “likely” term is used to define certainty greater than 90% of a variant either being disease-causing or benign.

### Pathogenic Variant

If the previously identified or novel variation has substantial evidence that it causes a disease with a known or unknown mechanism, is called a “pathogenic” variant. These kinds of variations are generally nonsense mutations, frame shift variations, or splice site alterations.

### Likely Pathogenic Variant

If the previously identified or novel variation is consistent with the diagnosis, it exists in the conserved genomic region, functional studies showed impaired gene product, or the function of the gene is known to be associated with a specific phenotype, the variation is called a “likely pathogenic” variant.

### Uncertain Significance Variant

If a variant cannot be classified as pathogenic or benign, it is called a “variant of uncertain clinical significance” (VUS). It can be a missense variation, an in-frame deletion, or an insertion. These kinds of novel variations can cause confusion during interpretation and reporting. If there is no other variant identified, VUS should be highlighted in the report.

### Likely Benign Variant

If a variant presents at high frequency in random individuals and is not a high penetrant or a disease-causing variant, it is called a “likely benign” variant. There is no absolute frequency threshold to classify that a variant is likely benign or likely pathogenic. This depends on the disease model, clinical characteristics, etc. These can be novel or previously reported variations with possible neutral effects. Generally, likely benign variants have enough evidence that they are not the cause of the disease, and the segregation analysis of haplotypes in affected and unaffected family members can support this finding.

### Benign Variant

If a previously reported variation is present at a higher frequency in the general population, it is called a “benign” variant. These variations are nonpathogenic and have neutral effects.

American College of Medical Genetics and Genomics suggested additional criteria including very strong, strong, and moderate support for being pathogenic, and likely pathogenic and likely benign variations.

Nonsense mutations, frame shifts, exonic deletions, and promoter variations (very strong) are generally assumed to cause loss of function in the genes. These kinds of variations lead to reduced or absent gene function and nonsense-mediated decay of an altered transcript. These kinds of variations are expected to affect the clinical findings.

Splice site variations may cause exon skipping and shortening or inclusion of intronic material due to loss or recreation of donor/acceptor splice sites. These kinds of variations are predicted to lead to a null effect that needs additional functional analysis (RNA or protein).

A missense variation is mostly known to be pathogenic; it alters the protein function or the nucleotide change and may disrupt the splice site. It can be detected by in silico prediction tools and then concluded to be a disease-related variation. Missense variations should be evaluated with minor allele frequency (MAF) values, which refer the second most common allele that occurs in a given population. The MAF value provides information to differentiate between common and rare variants in the population. If the determined missense variation has a low MAF value (<0.5%), it might be evaluated as a disease-related variation.

Although an index case might have the variation that is supporting the disease association, if the parents do not have it, it can be concluded as a “de novo” variation. However, in all cases, a detailed family history and verification of paternity is needed.

Furthermore, due to the germline mosaicism possibility, the same disease may affect more than one sibling. If there is only one affected proband and no previous history in a family, scientists should consider sequencing the unaffected parents of the proband to identify de novo mutations.

Another issue to be aware of is “compound heterozygosity”, and especially for autosomal recessive inherited disorders it should be carefully analyzed. Paternal validation is needed to understand the genetic background of different variations within the same gene, which come from the mother and the father of the index case.

## 4.1. DATABASE SEARCH: POPULATION- AND DISEASE-BASED

There are large numbers of databases in use, including both population-based and disease-based databases. Eliminating known variants that are present in public (dbSNP) and in-house variant databases and published projects such as the 1000 Genomes Project [28], EXAC, and the Exome Sequencing Project (ESP6500) [29] is a very helpful strategy to reduce the candidate list of disease-related variations. Population-based databases (such as the 1000 Genomes Project or ESP) have been created both for large and small local populations [30,31]. They are useful to obtain the frequencies of the variations. Disease databases mainly contain the variants of a specific disease or phenotype [32].

There are some limitations to these databases. For example, there is no absolute frequency threshold for a given variant, many populations are not represented, and there is no information about the phenotype. Limited numbers of locus-specific databases also exist but those are not available for most genes, there are contradictory data between databases, and they may not be updated. For correct data interpretation, researchers should check the updates of the databases, confirm that HGVS nomenclature is being used, and read the relevant publications [16,33]. Gene- or disease-oriented biomedical information can be found from the OMIM website (Online Mendelian Inheritance in Man- http://www.omim.org), in published scientific articles (PubMed- http://www.ncbi.nlm.nih.gov/pubmed), and in mutation databases (HGMD, Human Gene Mutation Database- http://www.hgmd.cf.ac.uk/ac/index.php).

Clinicians can interpret a variant when it is reported and track the genotype-phenotype correlation, population frequency of the variation, and clinical assertions. Clinical laboratories should increase their collaboration with clinicians to better understand the effect of the variation on the phenotype. The ClinVar database (http://www.ncbi.nlm.nih.gov/clinvar/) archives reports of the relationships among medically important human variations and phenotypes. It has access to dbSNP and dbVar and includes information about the location of variation and phenotypic descriptions included in MedGen (http://www.ncbi.nlm.nih.gov/medgen). ClinVar is an interactive tool that can be divided into submitter, variation, phenotype, interpretation, and evidence. ClinVar represents the interpretation of a single allele, compound heterozygotes, haplotypes, and combinations of alleles in different genes [34,35].

Searching for previously published scientific and medical studies is also a valuable tool for the annotation of a detected variant. Researchers should be aware of using older versions of nomenclature in published reports. The given information about the index case, affected family members, and the size of the family should be carefully noted to avoid incorrect data.

## 4.2. IN SILICO FUNCTIONAL PREDICTION

A variety of algorithms (SIFT, PolyPhen, Provean, CADD, Condel, GERP, SNAP, SNPs&Go, PhyloP, and MutationTaster) are used to determine the effect of variations and that can be done at the nucleotide, amino acid, protein, and transcript/splice variant levels (Table 1). Mainly they have been developed to estimate the deleterious effect of a variant on a protein. The most common use of these tools is to predict the impact of a missense variation on a protein and to predict the effect of the variation on splicing. The prediction depends on the location, evolutionary conservation, amino acid charge, 2D and 3D calculations of the effect on protein structure, and biochemical consequences of the amino acid substitution. Some of these tools are used for the prediction of the effects on splicing and loss or creation of the splice sites. As a limitation of in silico tools, variable and incompatible interpretation results are derived from different algorithms and the use of multiple programs is recommended because of the differing performances of the tools [36].

## 4.3. CLINICAL INTERPRETATION AND REPORTING OF NEXT-GENERATION SEQUENCING RESULTS

Interpretation and reporting of candidate genetic variations is the biggest challenge in NGS data analysis and reporting processes. Genetic testing based on WGS often results in several variations that are not directly clinically actionable. The reportable variations should be classified as pathogenic (P), likely pathogenic (LP), a variant of uncertain significance (VUS), likely benign (LB), or benign (B) as described by the ACMG.

Misinterpretation of data may be due to annotation errors, analytical errors, ethnicity effects (differential MAF values), reduced reproducibility in consideration of low-level mutations, nomenclature or terminology differences, and variable databases. International guidelines and recommendations developed to standardize and regulate deep sequencing are good references for researchers and clinicians [37].

Some general recommendations are summarized below to exclude possible incidental findings and ensure correct clinical interpretation and reporting of NGS results.

### Data Quality (Base Calling and Mapping)

- If the Q score is low (Q30 score is lower than 70%) and total coverage is lower than 80%, sequencing should be repeated.

- Different algorithms generate different outputs. Since the accuracy of the annotation depends on the success of the mapping, it is best to use at least two algorithms for mapping.

- Previous NGS data generated from the same laboratory (in-house data) are valuable to evaluate and exclude variations that arise from technical effects or poor quality of amplicon design. In-house laboratory data provide simplified analysis to exclude false variants (false positivity).

- Next-generation sequencing data aim to achieve a high diagnostic yield to achieve high coverage in all genomic regions covered. If genetic variation is detected by NGS with low coverage, resequencing should be repeated, and clear communication with the clinician is required if the test results cannot be used to exclude a particular clinical diagnosis.

### Reporting

- Next-generation sequencing results should not be transferred to clinical reports and practice without acceptable validation. It is essential to confirm the variation from a new DNA sample by NGS, Sanger sequencing, or another proper technique to exclude false positive results. Validation results should be included in the NGS report.

- All variants should be annotated and reported with regard to the gene name; gene symbol; heterozygous, homozygous, or compound heterozygous condition; nucleotide changes in coding regions; and amino acid changes in proteins according to the HGVS [16]. Mutalyzer is useful software to check the nomenclature for variations (https://mutalyzer.nl/). Each report should include the reference sequence and the use of unique nomenclature is critical; “g” represents the genomic sequence, “c” represents the coding sequence, “p” represents protein and “m” represents mitochondria, and the first translational codon (ATG) is the starting point. The universal reference genome (hg18, hg19, or hg38) and the latest versions should be used to give the correct genomic coordinates and it should cover the 5’ and 3′ untranslated regions and promoter regions (http://www.ncbi.nlm.nih.gov/refseq/) [38,39].

- Reports should state the limitations of each specific NGS test regarding the detection of different kinds of mutations.

- The reference genome, software, and databases (COSMIC, ClinVar, dbSNP, etc.) that are used should be specified in the report. If the variant was previously identified, the functional and clinical significance of the variation should be stated referring to the COSMIC database, the HGMD, or a scientific publication.

- For diagnostic purposes, only genes with a known (i.e. published and confirmed) relationship between the aberrant genotype and the pathology should be included in the analysis. The NGS test results should be included with the disease name, its targets, the names of the genes tested, their reportable ranges, the analytical sensitivity and specificity, and, if possible, the diseases not relevant to the clinical phenotype that could be caused by mutations in the tested genes [40].

- For diagnostic purposes, all pathogenic and likely pathogenic variants have to be reported. Whether or not variants of unknown significance (VUS) are reported will depend on local practice. Researchers should be very cautious if detailed laboratory analysis has not been performed and this should be included in the report. If no variation has been defined other than a VUS, it should be highlighted in the report. In that case, clinicians are strongly suggested to discuss the result with a clinical geneticist and it is acceptable to request additional analysis (parental testing, etc.) in order to facilitate the interpretation of the result (http://www.acgs.uk.com). The latter has to be clear for laboratory scientists, as well as for the referring clinicians [40].

## CONCLUSIONS

In medical use of genetic discoveries, it is quite important to improve the standards of data collection and sharing to define a systematic method for the clinical annotation and interpretation of genomic and phenotypic variations. Data-sharing platforms like the Undiagnosed Diseases Network (UDN- https://www.genome.gov) or Matchmaker Exchange Network (http://www.matchmakerexchange.org) for researchers of rare diseases and clinicians for sharing clinical phenotypes and sequencing data, which may allow for identification of other patients with the same phenotype, help us to understand the functional relevance of the variant that is obtained and reported [41,42].

Next-generation sequencing technology is being used as a diagnostic tool because of the expanded utility and reduced costs. Targeted sequencing offers better running times, costs, datasets, and coverage compared to WES or WGS. However, there are still many concerns about the application of NGS-based diagnostics. The challenges and clinical applications of NGS results have been discussed here. These include the accumulation and storage of huge amounts of genomic data, the need for bioinformatics experts, the need for national reference genomes, reimbursement of sequencing costs, and, of course, clinical interpretation of novel and VUS results.

**Glossary**

**Allele:** Alternative form of a given locus.

**Annotation:** DNA annotation or genome annotation is the identification of the locations of genes and all of the coding regions in a genome and determination of their function.

**Frameshift variation:** Genetic variation caused by indels (insertions or deletions) of a number of nucleotides in DNA.

**Missense variation:** A single nucleotide variation that leads to amino acid substitution and a codon change. Also called nonsynonymous substitution.

**Nonsense variation:** A single nucleotide variation that results in a premature stop codon, or a nonsense codon in the transcribed mRNA, and in a truncated, incomplete, and usually nonfunctional protein product.

**Deep sequencing:** Indicates that the total number of reads is many times larger than the length of the sequence under study.

**Depth:** In DNA sequencing refers to the number of times a nucleotide is read during the sequencing process.

**Coverage:** The average number of reads representing a given nucleotide in the reconstructed sequence.

**Whole-genome sequencing (WGS):** A laboratory process that determines the complete DNA sequence of an organism’s genome at a single time.

**Whole-exome sequencing (WES):** A technique for sequencing all the expressed genes in a genome (known as the exome).

**Amplicon (targeted) sequencing:** Amplicon sequencing refers to ultradeep sequencing of PCR products for analyzing genetic variations. Amplicon sequencing is a highly targeted approach for analyzing genetic variation in specific genomic regions.

**Pathogenic:** Anything that can produce disease.

**DNA polymorphism:** A heritable variation that is present in >1% of the population and increasingly detected by next-generation resequencing.

**Mutation:** DNA variants detectable in <1% of the population.

**Variation:** Now used for mutations and polymorphisms, a change in the DNA or RNA sequence compared to a reference genome.

**SNV:** Single nucleotide variation.

## Figures and Tables

**Table 1:**
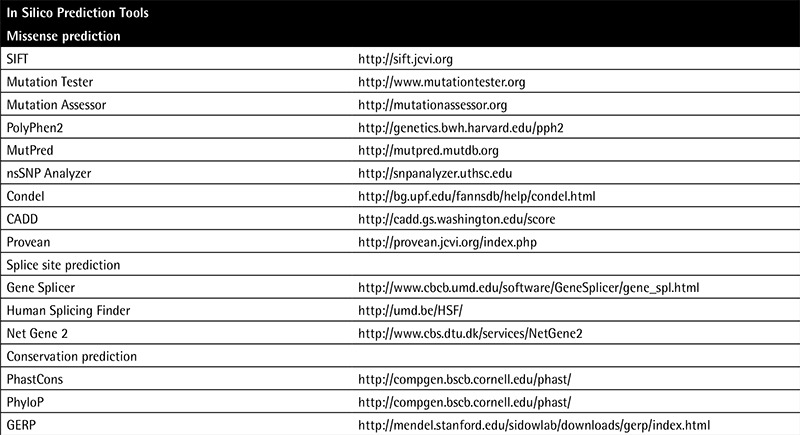
Representative in silico prediction tools and web pages.
